# Like me, like you – relative importance of peers and siblings on children’s fast food consumption and screen time but not sports club participation depends on age

**DOI:** 10.1186/s12966-020-00953-4

**Published:** 2020-04-15

**Authors:** Leonie H. Bogl, Kirsten Mehlig, Wolfgang Ahrens, Wencke Gwozdz, Stefaan de Henauw, Dénes Molnár, Luis Moreno, Iris Pigeot, Paola Russo, Antonia Solea, Toomas Veidebaum, Jaakko Kaprio, Lauren Lissner, Antje Hebestreit, on behalf of the IDEFICS and I. Family Consortia

**Affiliations:** 1 Leibniz Institute for Prevention Research and Epidemiology – BIPS, Achterstrasse 30, D-28359 Bremen, Germany; 2 Institute of Molecular Medicine FIMM, University of Helsinki, Helsinki, Finland; 3 Department of Epidemiology, Center for Public Health, Medical University of Vienna, Kinderspitalgasse 15, 1st floor, A-1090 Vienna, Austria; 4 School of Public Health and Community Medicine, Institute of Medicine, Sahlgrenska Academy, University of Gothenburg, Gothenburg, Sweden; 5 University of Bremen, Institute of Statistics, Bremen, Germany; 6 Department of Management, Society and Communication, Copenhagen Business School, Frederiksberg, Denmark; 7 Faculty Agricultural Sciences, Nutritional Sciences & Environmental Management, Justus-Liebig-University, Giessen, Germany; 8 Department of Public Health, Ghent University, Ghent, Belgium; 9 Department of Paediatrics, Medical School, University of Pécs, Pécs, Hungary; 10 GENUD (Growth, Exercise, Nutrition and Development) Research Group, Faculty of Health Sciences, University of Zaragoza, Zaragoza, Spain; 11 Institute of Food Sciences, National Research Council, Avellino, Italy; 12 Research and Education Institute of Child Health, Strovolos, Cyprus; 13 Department of Chronic Diseases, National Institute for Health Development, Tallinn, Estonia; 14 Department of Public Health, University of Helsinki, Helsinki, Finland

**Keywords:** Sibling pairs, Peer influences, Children, Adolescents, Fast food, Screen time, Sports club, Physical activity

## Abstract

**Background:**

Lifestyle interventions to prevent paediatric obesity often target family and peer settings; their success is likely to depend on the influence that peers and families exert on children’s lifestyle behaviors at different developmental stages.

**Objective:**

First, to determine whether children’s lifestyle behavior more closely resembles their peers’ or siblings’ behaviors. Secondly, to investigate longitudinally whether children’s behavioral change is predicted by that of their peers or their siblings as they grow older.

**Methods:**

The European prospective IDEFICS/I.Family cohort (baseline survey: 2007/2008, first follow-up: 2009/2010, and second follow-up: 2013/2014) aims at investigating risk factors for overweight and related behaviors during childhood and adolescence. The present investigation includes 2694 observations of children and their siblings aged 2 to 18 years. Peers were defined as same-sex, same-age children in the same community and identified from the full cohort. The longitudinal analysis (mean follow-up time: 3.7 years) includes 525 sibling pairs. Children’s lifestyle behaviors including fast food consumption (frequency/week), screen time (hours/week) and sports club participation (hours/week) were assessed by questionnaire. Data were analyzed using multilevel linear models.

**Results:**

Children’s lifestyle behavior was associated with the respective behavior of their peers and sibling for all 3 behaviors. For fast food consumption, the peer resemblance was more than 6-fold higher than the sibling resemblance and the peer resemblance surpassed the sibling resemblance by the age of 9–10 years. The similarities with peers for fast food consumption and screen time steadily increased, while the similarities with siblings steadily decreased with increasing age of the children (*P*_interaction_ < 0.001). In contrast, the relative importance of peers and siblings on sports club duration did not vary by the age of the children. Longitudinal results showed that children’s changes in fast food consumption were more strongly associated with those in their peer group than their sibling, in particular if the age gap between siblings was large.

**Conclusion:**

In conclusion, our results support the implementation of multi-setting interventions for improving lifestyle behaviors in children. Our findings might also guide future intervention studies in the choice of timing and setting in which interventions are likely to be most effective. From the ages of 9–10 years onwards, family- or home-based interventions targeting children’s fast food intake and screen time behavior may become less effective than school- or community-based interventions aimed at peer groups.

## Introduction

Children grow up in complex social environments, including families, peer groups, schools, and communities that all interact in their influence on children’s dietary intake, physical activity and sedentary behaviors [1, 2]. For young children, family members are the primary social context providing experiences and access to food and physical activity in which children develop healthy or unhealthy lifestyles. As children reach preschool and school age, they may modify their behavior as they spend more time away from home and encounter new social influences. Previous IDEFICS/I.Family research showed that younger siblings tended to be more alike in dietary intake than older siblings [3]. Consistent with this observation, twin studies have shown that the shared family environment is the predominant driver of dietary intake in young children [4], whereas this influence disappears by young adulthood [5, 6]. Likewise, exposures and experiences shared by family members have the strongest influence on children’s activity levels [7], but the home environment may become less important during adolescent years [8].

The transition from childhood to early adolescence represents another period marked by behavioral change, as youth spend more time with their peers [9], purchase more meals and snacks at fast food restaurants [10, 11] and decrease moderate to vigorous physical activity coupled with an increase in leisure-time computer use [12]. Previous studies have shown that adolescents’ snack intake [13, 14], fast food consumption [15, 16], physical activity levels [17] and sedentary behaviors [18] are correlated with those of their friends and peers. It is often assumed that peer influences are of increasing importance as children become older and more independent of their parents. However, studies on peer influences on children’s eating and physical activity behavior considering different developmental periods are currently lacking [19]. In addition, the literature on peers has largely developed independently from that of siblings, limiting our understanding of the relative importance of these two important social agents. The few studies that do exist and have examined siblings and friends simultaneously are small-scale and are qualitative in nature [20] or have focused on body mass index (BMI) [21] or substance use in children and adolescents [22, 23].

To fill these research gaps, we aimed to determine whether children’s dietary, sedentary and physical activity behaviors resemble those of their peers and sibling considering different developmental stages, operationalized by using the following variables: weekly consumption frequency of meals and snacks from fast food restaurants, weekly duration of screen time and weekly duration of sports club participation. We also aimed to investigate longitudinally whether children’s behavioral change is predicted by their peers’ and sibling’s change in the same behavior over time.

## Methods

### Participants

Data for this study were drawn from the IDEFICS/I.Family cohort, a prospective multi-center study aimed at investigating eating habits and lifestyle factors and how these affect the health of children and adolescents from the following eight European countries: Belgium, Cyprus, Estonia, Germany, Hungary, Italy, Spain and Sweden [24]. In each country, children were recruited in kindergartens and primary schools in their communities whose socio-demographic characteristics and infrastructure was typical for the region. A total of 16,228 children aged 2–9 years were recruited during the IDEFICS baseline survey (T0) in 2007/2008. Two years after baseline (T1), 13,596 children were examined of whom 11,041 had participated previously. The newly recruited 2555 children at T1 were from other schools or classmates of the T0 study participants. The same examination modules were deployed at T0 and T1. The observational design of the IDEFICS study was accompanied by a 2-year intervention programme for primary prevention of childhood obesity, which was implemented in school and community settings with follow-ups at T1 and T2. However, at T2 only information on the sustainability of the intervention with a self-completion questionnaire mailed to the parents of index children in the intervention regions was collected. As the starting point of the I. Family study (T3), another follow-up examination was conducted in 2013/2014 when the children who originally participated at T0/T1 (i.e. index children) were between 5 and 17 years. The participation proportion of children already examined at T1 was 52% [25]. In addition, siblings of about the same age range as the index children as well as parents were encouraged to participate in the I. Family study. Thus, the I. Family study examined 9617 children from 6167 families with an average of 2 children per family [24]. All applicable institutional and governmental regulations concerning the ethical use of human volunteers were followed during this research. Each center obtained ethical approval from the national/local ethics committees in accordance with the ethical standards of the 1964 Declaration of Helsinki and its later amendments. Children and their parents gave informed consent.

### Measures and variables

In the IDEFICS study, parents (or legal guardians) answered a self-administered questionnaire about their children’s health and lifestyle. In the I. Family study, most questionnaires were administered again, this time following an age-specific approach: for children up to the age of 11, parents proxy-reported their children’s information, while adolescents aged 12 years and above completed the questionnaire themselves. The number of household members below the age of 18 years was reported by a parent (or legal guardian) using self-completion questionnaires at T0 and at T1. In the I. Family study, the focus on entire families required the design of a new kinship interview, which was conducted by trained interviewers and enquired information on the family size and household composition [26].

Parental education was assessed as a proxy of socioeconomic status (SES) and reported by one of the parents. Education was coded country-by-country according to the International Standard Classification of Education (ISCED) [27] and then grouped as follows: low (levels 1 and 2), medium (levels 3 and 4) and high (levels 5 and 6). For the analyses, the maximum ISCED level of either parent was used and low and medium levels were combined.

### Outcome and predictor variables

The consumption of meals or snacks from fast food restaurants was assessed by the following two questions: ‘How many times does your child/do you eat in a fast food restaurant or at stands or kiosks to consume a full meal alternative to a main meal (breakfast, lunch, dinner)?’ and ‘How many times does your child/do you eat in a fast food restaurant or at stands or kiosks to consume some food as snacks between meals? ’ with possible answer choices of ‘Never’, ‘Once a month or less often’, ‘Several times a month’, ‘1-2 times a week’ and ‘3 or more times a week’. The response frequencies were converted to weekly frequencies of consumption using the conversion factors 0, 0.2, 0.8, 1.5, and 4, respectively. These two items were summed to represent the overall consumption of either meals or snacks consumed at fast food restaurants. We refer to this behavior as fast food consumption.

Screen time duration, as a proxy indicator for sedentary time, was calculated from these two questions: ‘How long does your child/do you usually watch TV/video/DVD per day?’ and ‘How long does your child/do you usually sit at a computer/game console per day?’ separately reported for weekdays and weekend days. The answer categories ‘Not at all’, ‘less than 30 min. per day’, ‘30 min. to 1 hour per day’, ‘about 1-2 hours per day’, ‘about 2-3 hours per day’ and ‘more than 3 hours per day’ were recoded to daily frequencies with the conversion factors 0, 0.25, 0.75, 1.5, 2.5 and 4, respectively. To derive weekly frequencies, the weekday estimates were multiplied by 5 and the weekend estimates by 2 and then summed to derive total screen time per week.

Information on sports club participation, as a proxy variable for physical activity, was obtained from two questions: ‘Is your child/are you a member of a sports club?’ and ‘How much time does he or she/do you spend doing sports in a sports club per week?’ For those who reported to be a member of a sports club, the answers given in hours and minutes were converted into hours of sports club duration per week. Sports participation by questionnaire was positively associated with accelerometer-derived physical activity [28].

The target children’s lifestyle behavior was the outcome variable and their sibling’s and peer group’s behavior the predictor variable.

### Definition of peers

For each target child and for each study wave (T0, T1 and T3), we defined peers as any children who participated in the IDEFICS/I.Family cohort and who were of the same sex in the same community within an age range of 1 year (2–2.9, 3–3.9, etc., 16–16.9, 17–17.9) of the child, excluding the target child. This definition is in line with other studies that have used broader definition of peers, such as the average weight in a community or region [29–32]. In the original IDEFICS study, the community-oriented intervention programme was conducted in 24 communities in eight European countries. In all countries, two communities were selected, with the exception of Sweden (3 communities) and Italy (9 communities). The participating communities in the respective countries are listed in the eTable 1. For the purpose of the present study and the definition of the peer groups, the Italian communities were summarized into two communities (one urban and one predominantly rural), leaving in total 17 communities for the definition of peers (see eTable 1)*.* Thus, for each child, peers from the same community were defined as all same-sex and same-age children who provided data on behavioral variables of interest. The peers were identified from the full cohort (*n* = 32,174 observations of children with behavioral data used in this analysis from altogether 3 study waves). For each child, 41 peers were identified on average (range: 1–165 peers per child); values of the behavioral variables were averaged across all peers to represent the mean behavior of the peer group.

### Description of the analysis dataset

We included children below the age of 18 from 2-child families, who participated in one or more of the examination waves and for whom data on the behavioral variables of interest and covariates were available for both children in the family. Based on these inclusion criteria, the final analysis dataset consisted of 5388 observations of children from 2694 observations on sibling pairs; 1499 sibling pairs participated in only one of the study waves, 380 pairs in 2 study waves and 145 pairs in all 3 study waves. The sample also included 189 observations on twin pairs; zygosity of the twin pairs was not determined. One child was randomly assigned as the target child and the other child in the family as the sibling resulting in 2694 target children and their siblings. Sibling pairs providing data on 2 study waves (T0 and T3 or T1 and T3) contributed to the longitudinal analysis (*n* = 1050 observations of children from 525 sibling pairs) (Table 1).

**Table 1:** Definition of data sets and analysis

Model	N^a^	Target children	N^b^	Comment	Analysis	Table/Figure
1	5388	Randomly assigned child per family	2694	All children	CS	Table 3
1a	2795	Boys, randomly assigned	1387	Stratified by the sex of the target child	CS	Table 3
1b	2593	Girls, randomly assigned	1307		CS	Table 3
1c	2726	Randomly assigned child with a SS sibling	1363	Stratified by the sex of the sibling	CS	Table 3
1d	2662	Randomly assigned child with an OS sibling	1331		CS	Table 3
1e	2572	Randomly assigned child with a near-aged sibling	1286	Stratified by the age difference with the sibling (≤ 2.7 vs > 2.7 years)	CS	Table 3
1f	2816	Randomly assigned child with a much younger or older sibling	1408		CS	Table 3
1 g	1920	Randomly assigned child, < 7 years	959	Stratified by age groups of the target children (< 7, 7–8, 9–10 and ≥ 11 years)	CS	Figure 1
1 h	1201	Randomly assigned child, 7–8 years	585		CS	Figure 1
1i	1095	Randomly assigned child, 9–10 years	570		CS	Figure 1
1j	1172	Randomly assigned child, ≥11 years	580		CS	Figure 1
2	1050	Randomly assigned child per family	525	All children	L	Table 4
2a	548	Boys, randomly assigned	272	Stratified by the sex of the target child	L	Table 4
2b	502	Girls, randomly assigned	253		L	Table 4
2c	530	Randomly assigned child with a same-sex sibling	265	Stratified by the sex of the sibling	L	Table 4
2d	520	Randomly assigned child with an opposite-sex sibling	260		L	Table 4
2e	380	Randomly assigned child with a near-aged sibling	190	Stratified by the age difference with the sibling (≤ 2.7 vs > 2.7 years)	L	Table 4
2f	670	Randomly assigned child with a much younger or older sibling	335		L	Table 4

^a^Number of observations on children from 2-child families

^b^Number of target children (= number of observations)

*CS* cross-sectional, *L* longitudinal

### Statistical analyses

Data preprocessing and statistical analyses were performed with Stata 15.1 (Stata Corporation, College Station, TX, USA, http://www.stata.com). Descriptive characteristics of the children from 2-child families are shown for the total sample and by age groups.

Data were analyzed using linear multilevel models (−mixed- in Stata) with the target children’s behavior as the dependent variable and the sibling’s and average peer group’s behavior as independent variables. Analyses were adjusted for age and sex of the target children, sibling age, sibling pair sex (same-sex vs. opposite-sex), birth order and parental education. Country and family ID were included as random effects to account for the nesting of children within countries and repeated measures of sibling pairs. Potential interactions were formally tested for sex of the target children, sex of the sibling pair, age differences between siblings and children’s age groups in the main model by including interaction terms. Interactions with a *p*-value below 0.01 were considered significant. All analyses were stratified by the sex of the target children, sibling pair sex and age groups of the target children (< 7 years, 7–8 years, 9–10 years, ≥11 years).

For those sibling pairs with longitudinal data, we computed the change in children’s behavior, sibling’s behavior and peer group’s behavior by calculating the difference between T0 (2007/2008) and T3 (2013/2014). For children with missing baseline information, T1 values were used as baseline values. To analyze whether the change in children’s behavior was predicted by the change in the same behavior of their sibling or peers, a similar multilevel model was used as described above and adjusted for the time interval between baseline and follow-up. Longitudinal analyses were adjusted for age and sex of the target children and siblings at baseline, sibling pair sex, birth order and parental education at follow-up.

We performed main analysis and stratified analyses as outlined in Table 1. Due to the random assignment of one child as target child per sibling pair, there was no difference in the average age of the target children and their sibling (8.4 years for both). Stratified analyses of the main dataset were performed by sex of the target children (1a and 1b), by sex of the sibling (same vs opposite sex) (1c and 1d), by the age gap between siblings (≤ 2.7 years vs. > 2.7 years based on the median age difference) (1e-1f) and by age groups (1 g-1j). Finally, in the subset of sibling pairs for whom longitudinal data were available, similar analyses were performed for male and female target children (2a-b), for target children with same-sex and opposite-sex sibling (2c-d) and for target children with near-aged siblings or siblings with a larger age difference (2e-2f). Again, the mean age did not differ between the target children and their siblings (5.9 vs. 5.8 years at baseline) who provided longitudinal data.

## Results

Children’s characteristics are presented in Table 2, with nearly half of the sample being girls (48%). More than half of the children (54%) were from families with high parental education level. Children’s fast food consumption, screen time duration and sports club duration increased with age.

**Table 2:** Children’s characteristics for all children and by age group of the target children

	Observations of children^1^				
	All children	< 7 years	7–8 years	9–10 years	> = 11 years
**Observations of children from 2-child families (*n*)**	5388	1920	1201	1095	1172
**Number of peers per child (mean, SD)**	40.7 (22.9)	36.4 (22.6)	42.4 (25.6)	41.0 (19.6)	45.7 (22.3)
**Age (mean, SD)**	8.4 (3.2)	5.1 (1.3)	7.9 (0.6)	9.9 (0.6)	13.1 (1.4)
**Sex (n, %)**					
Boys	2795 (51.9)	1041 (54.2)	609 (50.7)	557 (50.9)	588 (50.2)
Girls	2593 (48.1)	879 (45.8)	592 (49.3)	538 (49.1)	584 (49.8)
**Parental education level (n, %)**^**2**^					
Low or medium education	2481 (46.1)	861 (44.8)	546 (45.5)	493 (45.0)	581 (49.6)
High education	2907 (53.9)	1059 (55.2)	655 (54.5)	602 (55.0)	591 (50.4)
**Country (n, %)**					
Italy	1084 (20.1)	350 (18.2)	247 (20.6)	229 (20.9)	258 (22.0)
Estonia	428 (7.9)	156 (8.1)	68 (5.7)	127 (11.6)	77 (6.6)
Cyprus	724 (13.4)	132 (6.9)	120 (10.6)	145 (13.2)	327 (27.9)
Belgium	476 (8.8)	231 (12.0)	134 (11.2)	74 (6.8)	37 (3.2)
Sweden	908 (16.9)	426 (22.2)	234 (19.5)	154 (14.1)	94 (8.0)
Germany	684 (12.7)	232 (12.1)	152 (12.7)	124 (11.3)	176 (15.0)
Hungary	528 (9.8)	146 (7.6)	111 (9.2)	121 (11.1)	150 (12.8)
Spain	556 (10.3)	247 (12.9)	135 (11.2)	121 (11.1)	53 (4.5)
**Children**’**s lifestyle behavior**					
Fast food consumption (frequency/week)	1.02 (1.66)	0.44 (0.67)	0.47 (0.68)	0.62 (1.00)	2.92 (2.43)
Screen time duration (hours/week)	13.7 (8.8)	10.6 (6.6)	13.2 (7.2)	14.6 (8.1)	18.4 (11.5)
Sports club duration (hours/week)	1.79 (2.22)	0.85 (1.29)	1.94 (1.92)	2.48 (2.24)	2.53 (3.01)
**Peer**’**s lifestyle behavior**					
Fast food consumption (frequency/week)	1.03 (1.27)	0.45 (0.29)	0.51 (0.34)	0.59 (0.41)	2.95 (1.51)
Screen time duration (hours/week)	13.9 (4.3)	10.9 (2.7)	13.5 (2.5)	14.7 (3.0)	18.5 (4.6)
Sports club duration (hours/week)	1.69 (1.02)	0.78 (0.59)	1.83 (0.60)	2.39 (0.77)	2.36 (1.03)

^1^Pooled sample from 3 study waves

^2^International Standard Classification of Education Maximum (ISCED); maximum of both parents (0, 1, 2 = low education; 3, 4 = medium education; 5, 6 = high education)

### Cross-sectional results (multilevel linear models)

For all 3 behaviors, children’s behaviors were associated with the respective behaviors of their siblings and peers (Table 3). For fast food consumption and sports club duration, children’s behaviors were more strongly associated with that of their peers than their sibling, while for screen time duration, the peer and sibling effects were about similar in magnitude. For fast food consumption, the peer estimate was more than 6 times as large as the sibling estimate. The associations between children’s screen time and sports club duration with the respective peer behaviors were stronger in boys than in girls (P_interaction_ < 0.01). The associations did not vary by the sex of the sibling (whether of the same or opposite-sex), except for screen time duration, for which same-sex siblings were more similar to one another (P_interaction_ = 0.001). For all 3 behaviors, near-aged siblings (≤ 2.7 years) were more similar to one another than siblings with a larger age difference (> 2.7 years). In addition, the resemblance with peers was lower in children with a near-aged sibling than in children with a sibling of greater age difference (P_interaction_ < 0.001 for both).

**Table 3:** The relative importance of peer and sibling behavior for all children, and stratified by target children’s sex, sibling pair sex and age difference between siblings

				Fast food consumption (frequency/week)		Screen time duration (hours/week)		Sports club duration (hours/week)	
**Model**	**Outcome: child’s behavior**	**N**	**Explanatory variables**	**β**	**95% CI**	**β**	**95% CI**	**β**	**95% CI**
1	All children	2694	Peer’s behavior	0.89	0.85, 0.94	0.51	0.42, 0.61	0.54	0.45, 0.64
			Sibling’s behavior	0.14	0.11, 0.17	0.46	0.43, 0.49	0.34	0.31, 0.37
1a	Boys	1387	Peer’s behavior	0.84	0.77, 0.91	0.53	0.40, 0.67	0.53	0.39, 0.68
			Sibling’s behavior	0.12	0.08, 0.16	0.47	0.42, 0.52	0.33	0.28, 0.37
1b	Girls	1307	Peer’s behavior	0.94	0.88, 1.01	0.31	0.17, 0.44	0.45	0.31, 0.59
			Sibling’s behavior	0.17	0.13, 0.22	0.48	0.44, 0.52	0.36	0.31, 0.40
1c	Children with a same-sex sibling	1363	Peer’s behavior	0.92	0.86, 0.98	0.49	0.37, 0.62	0.50	0.36, 0.63
			Sibling’s behavior	0.17	0.13, 0.21	0.52	0.48, 0.56	0.35	0.31, 0.40
1d	Children with an opposite-sex sibling	1331	Peer’s behavior	0.87	0.80, 0.94	0.49	0.35, 0.63	0.59	0.45, 0.73
			Sibling’s behavior	0.11	0.06, 0.16	0.42	0.37, 0.47	0.33	0.28, 0.37
1e	Children with a near-aged sibling	1286	Peer’s behavior	0.78	0.71, 0.84	0.34	0.22, 0.47	0.43	0.29, 0.56
			Sibling’s behavior	0.22	0.18, 0.26	0.59	0.55, 0.63	0.48	0.44, 0.53
1f	Children with a much younger or older sibling	1408	Peer’s behavior	0.94	0.87, 1.01	0.62	0.49, 0.75	0.55	0.41, 0.68
			Sibling’s behavior	0.10	0.05, 0.15	0.37	0.33, 0.41	0.26	0.22, 0.30

N, number of observations of children

*P* < 0.001 for all

Each child from a sibling pair was randomly assigned to be the target child or sibling

Linear mixed models adjusted for age and sex of the target children, age of the sibling, sex of the sibling pair, birth order, parental education, and country and family ID as random effects

In model 1e the age difference between the sibling is ≤2.7 years. In model 1f the age difference between siblings is > 2.7 years

**Fig. 1:**
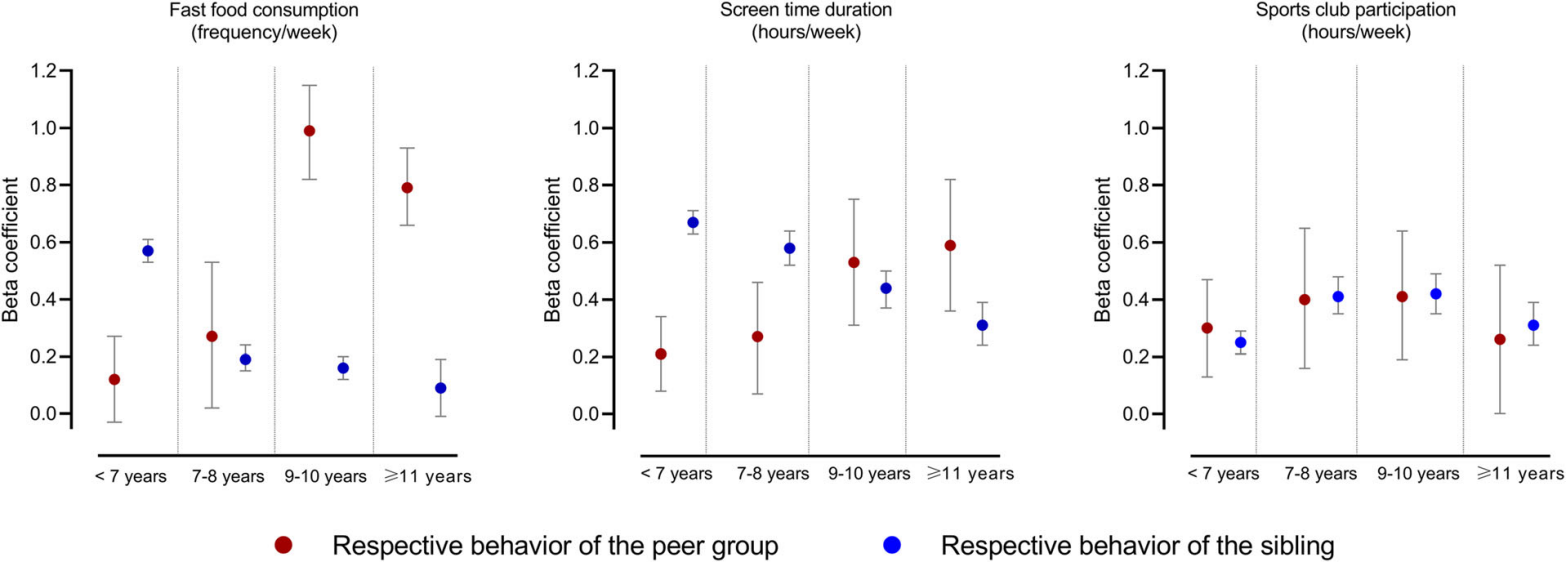
The relative importance of peer’s and sibling’s behavior on children’s fast food consumption, screen time and sports club duration by age group of the children. Linear mixed models adjusted for age and sex of the target children, age of the sibling, sex of the sibling pair, birth order, parental education, and country and family ID as random effects (Models 1 g-1j). Each child in a sibling pairs was randomly assigned to be the target child or sibling

Figure 1 shows that for fast food consumption and screen time duration, younger children’s behavior was more strongly associated with that of their sibling than peers, while older children’s behavior was more strongly associated with that of their peers than sibling. Children’s fast food consumption was not associated with that of their peers in children below the age of 7 years. Thereafter, the peer resemblance steadily increased until ages 9–10 years and remained high in the oldest age group (≥ 11 years). The sibling resemblance for fast food consumption steadily decreased over the age groups and became non-significant in children ≥11 years. Screen time duration of children was associated with that of their siblings and peers in all age groups and similar to fast food consumption, the peer resemblance steadily increased and the sibling resemblance decreased with increasing age of the children. No clear trends over the age groups were observed for sports club duration. Peers’ sports club duration was not a significant predictor of sports club duration in children ≥11 years. The interaction terms for ‘peer behavior x age group’ and ‘sibling behavior x age group’ proved highly significant for fast food consumption and screen time duration (P_interaction_ < 0.001).

### Longitudinal results (multilevel linear models)

In total, similarities of behavior change was examined in 1050 children from 525 sibling pairs. The children were 5.9 years (range, 2.0–11.1 years) at baseline and 9.5 years (range, 3.9–15.2 years) at follow-up. The mean follow-up time was 3.7 years (range, 1–8 years). We investigated whether the change in children’s behaviors was predicted by the change in the same behavior of their sibling and peer group. Longitudinal results were largely similar to cross-sectional findings and are presented for the whole sample of children and stratified sample in Table 4. In the overall sample, children’s change in fast food consumption was more strongly predicted by that of their peers than that of their siblings. However, this association varied between children who have a sibling close versus a sibling more distant in age. In children with a near-aged sibling, the change in fast food consumption was predicted by both their near-aged sibling and their peers in about equal magnitude. In contrast, in children whose sibling was more than 2.7 years apart in age, the peer resemblance was about 9 times greater than the sibling resemblance (Table 4). For screen time and sports club duration, children’s behavioral change was predicted by that of their siblings and peers, and associations were of about equal strength.

**Table 4:** The relative importance of peers and sibling behavior change on children’s lifestyle behavioral change  for all children, and stratified by target children’s sex, sibling pair sex and age difference between siblings

				Fast food consumption (frequency/week)		*P*-value	Screen time duration (hours/week)		*P*-value	Sports club duration (hours/week)		*P*-value
Model	Outcome: child’s behavior	N	Explanatory variables	β	95% CI		β	95% CI		β	95% CI	
2	All children	525	Peer’s behavior change	0.93	0.81, 1.04	< 0.001	0.46	0.24, 0.68	< 0.001	0.43	0.18, 0.68	0.001
			Sibling’s behavior change	0.14	0.07, 0.21	< 0.001	0.40	0.33, 0.47	< 0.001	0.28	0.20, 0.36	< 0.001
2a	Boys	272	Peer’s behavior change	0.97	0.81, 1.12	< 0.001	0.55	0.25, 0.84	< 0.001	0.33	0.01, 0.64	0.041
			Sibling’s behavior change	0.14	0.06, 0.22	0.001	0.44	0.34, 0.54	< 0.001	0.23	0.13, 0.33	< 0.001
2b	Girls	253	Peer’s behavior change	0.87	0.70, 1.04	< 0.001	0.12	−0.25, 0.48	0.530	0.56	0.16, 0.96	0.007
			Sibling’s behavior change	0.12	0.00, 0.24	0.052	0.39	0.30, 0.49	< 0.001	0.34	0.21, 0.47	< 0.001
2c	Children with a same-sex sibling	265	Peer’s behavior change	0.91	0.75, 1.07	< 0.001	0.51	0.20, 0.82	0.001	0.44	0.09, 0.79	0.013
			Sibling’s behavior change	0.23	0.14, 0.33	< 0.001	0.50	0.40, 0.61	< 0.001	0.28	0.16, 0.41	< 0.001
2d	Children with an opposite-sex sibling	260	Peer’s behavior change	0.94	0.78, 1.11	< 0.001	0.34	0.04, 0.64	0.032	0.35	−0.02, 0.72	0.062
			Sibling’s behavior change	0.03	−0.06, 0.13	0.487	0.32	0.23, 0.42	< 0.001	0.26	0.15, 0.38	< 0.001
2e	Children with a near-aged sibling (≤ 2.7 years differences)	190	Peer’s behavior change	0.48	0.25, 0.72	< 0.001	0.69	0.30, 1.09	0.001	0.44	0.09, 0.79	0.013
			Sibling’s behavior change	0.41	0.15, 0.68	0.001	0.52	0.40, 0.64	< 0.001	0.28	0.16, 0.41	< 0.001
2f	Children with a much younger or older sibling (> 2.7 years differences)	335	Peer’s behavior change	1.06	0.90, 1.21	< 0.001	0.25	−0.02, 0.51	0.065	0.35	−0.02, 0.72	0.062
			Sibling’s behavior change	0.11	0.05, 0.19	0.001	0.33	0.25, 0.41	< 0.001	0.26	0.15, 0.38	< 0.001

N, number of observations of children

Each child from a sibling pair was randomly assigned to be the target child or sibling

Linear mixed models adjusted for age and sex of the target children, age of the sibling, sex of the sibling pair, birth order, parental education, and country and family ID as random effects

In model 1e the age difference between the sibling is ≤2.7 years. In model 1f the age difference between siblings is > 2.7 years

## Discussion

In this sample of European children and adolescents, we found that children’s lifestyle behavior was associated with the respective behavior of their peers and siblings. The novel aspect of the present study was the joint investigation of peer and sibling influences in the same population considering different behaviors and different developmental stages.

It is often assumed, but not established, that familial influences decrease and peer influences increase as children become older and more independent from their parents. For screen time behavior and fast food consumption, the peer resemblance steadily increased from 7 to 11 years, while the sibling resemblance steadily decreased with increasing age of the children. For fast food consumption, children below the age of 7 years showed no peer resemblance, while children 11 years and above years no longer resembled their siblings. Longitudinal findings largely confirmed the cross-sectional associations, showing that with increasing age, children’s change in fast food consumption strongly resembles that of their peers and to a lesser extent that of their sibling. However, we also found that near-aged siblings were more similar in behavioral change than siblings with a larger age gap between them. In particular, for fast food consumption, we observed that when children have a sibling who is closer in age, the sibling similarity is stronger and the peer similarity is weaker as compared to when children have a sibling with a greater age difference. To a large extent, younger children depend on the accessibility of foods at home and screen time is generally a home activity that is bound by family rules [33]. With increasing age, children spend more unsupervised time with their friends [9] and they often receive pocket money that is frequently spent on soft drinks and fast food [34, 35] outside the home environment [36]. Family members are more alike in the intake of healthy than unhealthy foods [3], while the opposite is true for friends [37], and friends tend to be particularly similar for snack and fast food consumption [13–16, 38].

With increasing age, children might become more susceptible to peer pressures and peer social norms shaping their dietary and physical activity behavior. Generally, physical activity significantly declines during adolescence [39] (10–19 years old). As an example, being teased by peers and image concerns have been mentioned by a number of girls as to why they ceased participation in sports and other physical activities during adolescence [40]. Food or eating style may have symbolic and social meaning and junk food is associated with coolness [41] and regarded as the normative behavior by other teenagers [42]. Consequently, fitting in the peers’ norms [43] may be an explanation for our observation of a steady increase in relative child-peer resemblance for fast food consumption and screen time with increasing age. In the present study, the resemblance for fast food with peers surpassed the resemblance with siblings significantly by the age of 9–10 years.

We found some indication of gender differences, with greater peer resemblances for weekly screen time duration and sports club duration for boys than girls. A previous social network analysis that assessed peer status through friendship nomination also found higher correlations between friend’s physical activity and screen time for boys than girls [44]. We also observed that same-sex siblings were more alike in screen time behavior than opposite-sex siblings, but same-sex siblings were no more similar than opposite-sex siblings in fast food consumption or sports club participation. In our previous study based on I. Family data, sibling resemblances in food group intake for sister–sister, sister–brother, and brother–brother pairs were about similar in magnitude [3]. Together these results suggest that siblings of the same and opposite sex experience rather similar social environments for eating, but not for screen time, for which siblings of the same sex resembled one another more than opposite-sex siblings.

We used a broad definition of peers that might include friends, acquaintances, classmates and sport mates but also unfamiliar peers of the child. In this sense, our broad definition of peers represents the extra-familial environment shared among children of the same sex and age who grow up in the same community. Because of this broad definition of peers, our findings are not directly comparable with studies that examined the influence of friends by friendship nominations. Other studies have used similar broad definitions of peer groups at the country-level [32], community-level [45] or school-level [22, 30]. Children grow up with their peers and siblings in the same communities, and both types of relationships are therefore exposed to the same neighborhood effects, as well as similar societal and cultural values regarding lifestyle, e.g. dietary or media behavior. Correlations between children who are neighbors provide upper-bound estimates of the extra-familial context shared by peers including neighborhoods, communities, schools and peers [46]. As siblings share both the family environment and the neighborhood, sibling correlations represent upper-bound estimates of the familial context, that includes familial (including shared genes) and neighborhood influences on children’s behavior [22]. Thus, our data provide important insight into the degree to which different lifestyle behaviors are explained by extra-familial (peers) and familial contexts (siblings) in school-aged children in Europe.

The strengths and limitations of this study design have to be addressed. As very few large families participated in the IDEFICS/I.Family cohort, we restricted the analysis group to two-child families; thus, our findings are not readily generalizable to larger families. Our data do not permit a detailed examination of self-selection, the shared built environment and social contagion as possible explanations for the observed sibling and peer resemblances. Evidence form other studies that examined BMI suggest the presence of social contagion in obesity [47, 48]. A further limitation is the lack of data on new technologies such as the use of touchscreen tablets and smartphones by children. The strengths include the large sample of European children and adolescents from 8 European countries that followed a standardized protocol and completed a detailed interview on kinship and household. This unique dataset facilitated the examination of the relative importance of peer of sibling behavior on children’s lifestyle behavior in the same sample, addressing also sex and age differences. Cross-sectional and longitudinal results were in line, which further supports the robustness of our results.

In summary, children’s fast food consumption, screen time behavior and sports club participation were associated with the respective behaviors of their peer group and their sibling. For fast food consumption, the peer resemblance was more than 6-fold higher than the sibling resemblance, and the peer resemblance surpassed the sibling resemblance by the age of 9–10 years. Similarly, for screen time behavior, the peer resemblance steadily increased while the sibling resemblance decreased from the age of > 7 years onwards. Our results support the design and implementation of future multi-setting intervention studies that target, for example, schools, families and neighborhoods simultaneously in order to improve lifestyle behaviors of children. Our findings further suggest that as children grow older, family- or home-based interventions might become less effective as compared to school- or community-based interventions aiming at peer groups. Future research may evaluate whether healthy school environments and neighborhoods foster healthy behaviors in pre-adolescence and adolescence, in particular in places where young people eat, hang out with their friends and where they engage in physical activity.

## Supplementary information

**Additional file 1.** eTable 1 IDEFICS/I.Family communities in 8 European countries

## Data Availability

Due to the highly sensitive data collected in children, ethical restrictions prohibit the authors from making the minimal data set publicly available. Data are however available from the authors upon reasonable request and with permission of the Steering Committee on a case-by-case basis. Interested researchers can contact the I. Family consortium (http://www.ifamilystudy.eu/) or the study co-ordinator (Ahrens@leibniz-bips.de) to discuss possibilities for data access.
