# Supplementary material for: Like me, like you – relative importance of peers and siblings on children’s fast food consumption and screen time but not sports club participation depends on age
Source: Int J Behav Nutr Phys Act. 2020 Apr 15;17:50. doi: 10.1186/s12966-020-00953-4 (PMC7160987; doi:10.1186/s12966-020-00953-4)
Supplement: Supplementary file 1 — Additional file 1. eTable 1 IDEFICS/I.Family communities in 8 European countries [file 12966_2020_953_MOESM1_ESM.docx]

**eTable 1. IDEFICS/I.Family communities in 8 European countries**

| **Country** | **Communities** | **Observations of children** | **Mean number of peers per child** | **Distance between them (km)** |
| --- | --- | --- | --- | --- |
| Belgium | 1. Geraardsbergen  2. Aalter | 214  252 | 43  40 | 46 |
| Cyprus | 1. Strovolos  2. Pafos | 438  270 | 48  40 | 120 |
| Estonia | 1. Tartu  2. Tallinn | 184  228 | 40  41 | 185 |
| Germany | 1. Delmenhorst  2. Wilhelmshaven | 404  266 | 39  34 | 68 |
| Hungary | 1. Pecs  2. Zalaegerszeg | 252  268 | 43  37 | 205 |
| Italy | 1. Avellino center, Avellino outskirt, Atripalda, Mercogliano, Monteforte  2. Pratola Serra, Volturara Irpina, Forino, Lauro/Quindici | 754  310 | 55  34 | 5- 43 |
| Spain | 1. Huesca  2. Zaragoza (1st district) | 284  252 | 34  36 | 72 |
| Sweden | 1. Partille  2. Alingsas  3. Mölndal | 496  300  100 | 39  30  23 | 16-38 |
